# Unique Characteristics of Epilepsy Development in Neurocysticercosis

**DOI:** 10.4269/ajtmh.19-0485

**Published:** 2020-05-18

**Authors:** Jesica A. Herrick, Javier A. Bustos, Philip Clapham, Hector H. Garcia, Jeffrey A. Loeb, for the Cysticercosis Working Group in Peru

**Affiliations:** 1 Division of Infectious Diseases, Immunology, and International Medicine, University of Illinois at Chicago, Chicago, Illinois;; 2 Center for Global Health, Instituto Nacional de Ciencias Neurológicas, Universidad Peruana Cayetano Heredia, and Cysticercosis Unit, Lima, Perú;; 3 Department of Neurology and Rehabilitation Medicine, University of Illinois at Chicago, Chicago, Illinois

## Abstract

The parasitic helminth infection neurocysticercosis (NCC) is the most common cause of adult-acquired epilepsy in the world. Despite the serious consequences of epilepsy due to this infection, an in-depth review of the distinct characteristics of epilepsy due to neurocysticercosis has never been conducted. In this review, we evaluate the relationship between NCC and epilepsy and the unique characteristics of epilepsy caused by NCC. We also discuss recent advances in our understanding of NCC-related epilepsy, including the importance of anti-inflammatory therapies, the association between NCC and temporal lobe epilepsy, and the recent discovery of biomarkers of severe epilepsy development in individuals with NCC and seizures.

## INTRODUCTION

The parasitic infection neurocysticercosis (NCC) is the most common etiology of adult-acquired epilepsy (an enduring predisposition for seizures) worldwide, associated with 30–50% of such cases of epilepsy in endemic areas.^1–12^ The most common clinical presentation of NCC is seizures, occurring in up to 80% of infected individuals with symptoms.

Seizures can be classified, according to the International League Against Epilepsy, as either focal- or generalized-onset seizures.^13^ Focal seizures can be further divided into aware or impaired awareness. As generalized-onset seizures by definition involve networks on both sides of the brain at onset, they are always associated with impaired awareness. Both focal- and generalized-onset seizures are then classified by the type of symptoms experienced at the onset of the seizure, either motor symptoms (e.g., tonic, clonic, and myoclonic) or non-motor symptoms (e.g., changes in sensation or cognition).

Regardless of the seizure type, epilepsy of any etiology is believed to develop in the setting of an epileptic abnormality that impairs normal neuronal activity, an altered seizure threshold, and/or additional factors.^14^ In the case of NCC, the epileptic abnormality is typically caused by focal lesions,^15^ although other chronic changes resulting from NCC infection (such as perilesional gliosis or areas of infarcts from infection-associated vasculitis) may disturb normal neuronal activity.^16,17^ Additional factors that may also play a role in epilepsy development for some patients with NCC include a genetic predisposition toward a lowered seizure threshold and/or a strong host immune inflammatory response.^18–20^ Although NCC is a leading cause of epilepsy, little attention has been paid to the distinct characteristics of NCC-related epilepsy. In this review, we examine the unique characteristics of epilepsy development in NCC.

When discussing seizures associated with NCC, it is important to distinguish early-onset seizures provoked by an active NCC infection and resulting inflammation (non-epileptic early-onset seizures) from epileptic seizures, which are unprovoked. Early-onset–provoked seizures are thought to be primarily incited by an immune inflammatory response to antigens released either from a naturally degenerating lesion or following treatment.^18,21^ These seizures may have multiple relapses associated with episodic lesion-associated inflammation. What distinguishes these acute seizures from epileptic seizures is that acute seizures do not continue in the absence of an active immune/inflammatory response to the parasite. It is only when this inflammatory response is also accompanied by the formation of synchronous firing patterns and epileptic networks where epileptogenesis (epilepsy development) occurs. Once these functionally connected epileptic networks form, it is unclear if they ever go away.

Factors associated with prior NCC infection can acutely lower the seizure threshold even in the absence of active infection. This can occur because of 1) neurotoxicity of calcium released from calcified lesions^22,23^ or 2) transient release of NCC antigen during remodeling of calcified lesions causing an immune inflammatory response. An inflammatory response to NCC calcifications was demonstrated in a histologic examination of two calcified lesions.^24,25^ In subjects who have formed epileptic neural networks, an acute lowering of the seizure threshold can lead to additional epileptic seizures. Various studies have shown that up to 30% of NCC patients with epilepsy who were successfully treated for NCC can have persistent seizures despite being treated with both antiparasitic and anticonvulsant medications.^18,21^ These recurrent seizures are associated with worsened quality of life and increased mortality.^21,26–35^

Epilepsy due to NCC has many distinct characteristics that may lead to clinical differences and altered optimal treatment strategies compared with epilepsy of other etiologies. Because this syndrome is common, it is worth evaluating these distinct characteristics. In this review, we discuss the features of epilepsy in subjects with NCC and review new advances in our understanding of NCC-related epilepsy.

### Clinical characteristics of epilepsy due to NCC.

**Figure 1.**
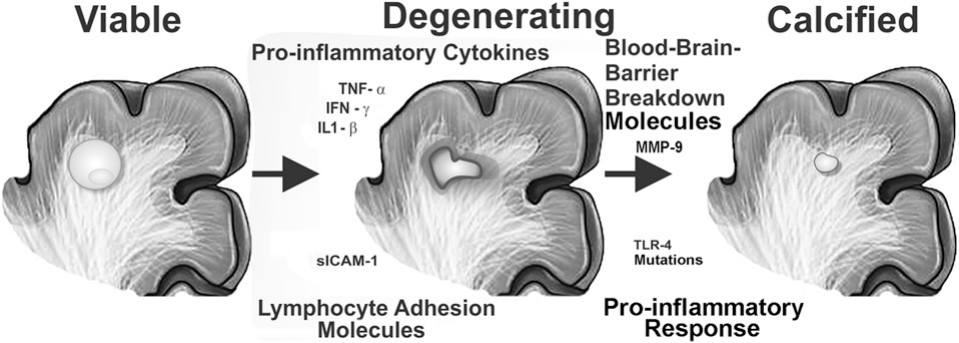
Inflammation-associated factors associated with epilepsy development in neurocysticercosis (NCC) patients. The figure summarizes the stages of NCC lesions: living viable cysts (left), degenerating dying lesions with inflammation (center), and dead calcified lesions (right). Markers that are increased in those with active epilepsy compared with NCC-infected subjects without epilepsy are shown: 1) pro-inflammatory cytokines (tumor necrosis factor-α, interferon-γ, and interleukin 1-β) and blood–brain barrier breakdown molecules (MMP-9); 2) lymphocyte adhesion molecules; and 3) mutations in regulators of lymphocyte adhesion (Toll-like receptor 4) associated with pro-inflammatory conditions.

Neurocysticercosis occurs when an individual is infected by the ova of *Taenia solium*, most commonly via the fecal–oral route. The infection passes through several stages that can be distinguished on imaging studies, from a living (viable) cyst, to an inflamed (degenerating) lesion, and finally culminates in the formation of a granuloma that either resolves (is no longer visible on imaging) or forms a calcified lesion (Figure 1).^23,36–38^ The clinical presentation of seizures in NCC patients is likely impacted by whether the patients have multiple or single lesions, new-onset seizures, or established epilepsy, and whether or not they have been treated with antiparasitic medications.^39,40^ Despite this clinical heterogeneity, multiple studies have confirmed that the most common seizure types associated with NCC are focal (occurring in 22–61% of NCC patients with new-onset seizures) and secondarily generalized-focal seizures (15–61% of subjects).^39,41^

Various studies have evaluated for an association between characteristics of NCC lesions and seizure semiology (clinical signs and symptoms), with mixed results. In some studies, the frontal lobe lesions were more likely to present with focal seizures, whereas the temporal lesions and those in the degenerating stage were more likely to secondarily generalize.^39,42–44^ In a retrospective cohort study, a strong relationship was found between the semiologic classification of the seizure and the location of the NCC lesion,^45^ but other studies have not found this association. One explanation for this discrepancy could be that different studies may have included distinct subpopulations of patients with NCC. Many of the studies which did not find an association between semiology and lesion characteristics included primarily calcified lesions, which represent subjects at a late stage of disease who thus may present differently.^46,47^ These conflicting results also further highlight the exceedingly heterogenous nature of NCC.

Several studies have demonstrated a benefit of antiparasitic treatment in reducing the frequency of seizures in subjects with NCC. In a double-blind, placebo-controlled trial of subjects with viable NCC lesions, treatment with albendazole and dexamethasone was shown to decrease the number of generalized seizures during 30 months of follow-up. The number of total seizures, however, was not decreased by treatment. Unsurprisingly, those who were treated with albendazole in this study were more likely to have resolved their cystic lesions on follow-up imaging than those randomized to placebo.^40^ This finding is especially important as a separate randomized controlled study found a significant reduction in seizures in subjects treated for NCC; this reduction was primarily driven by the subgroup of subjects for whom the cyst resolved.^48^ However, the authors noted that the cyst resolution only partially reduced the likelihood of continued seizures, and therefore, additional methods capable of preventing recurrent epileptic seizures in NCC patients need to be identified.

### Markers of increased risk for epilepsy development in patients with NCC.

Early intervention is thought to provide the best possibility for preventing epilepsy in subjects with an initial seizure, as each seizure may increase the brain’s ability to produce seizures.^49^ Unfortunately, regardless of the underlying cause of seizures, currently available anticonvulsant drugs do not prevent epileptogenesis or cure epilepsy.^50^ Therefore, for early intervention to prevent epilepsy in NCC patients to be possible, researchers must first 1) develop an increased understanding of the processes involved in epileptogenesis and 2) identify biomarkers capable of identifying subjects early in their clinical course who will go on to develop epilepsy. Such biomarkers would also help avoid unnecessary interventions in patients with a low likelihood of seizure recurrence and could identify novel therapeutic targets to prevent epilepsy.

**Figure 2.**
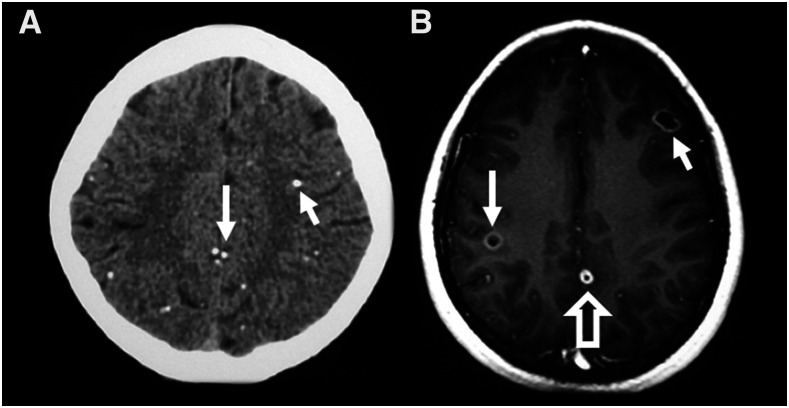
Burden of disease. An increased number of lesions may be associated with an increased likelihood for treatment-resistant seizures in subjects with NCC. (**A**) Axial computed tomography (CT) scan of a patient with NCC who had multiple calcified lesions. Representative calcifications on this image are indicated by solid arrows. (**B)** Axial T1 MRI scan demonstrating multiple degenerating lesions. The image shows two lesions in the colloidal stage of degeneration that are indicated with solid arrows; a lesion in the nodular stage is indicated with a hollow arrow.

**Figure 3.**
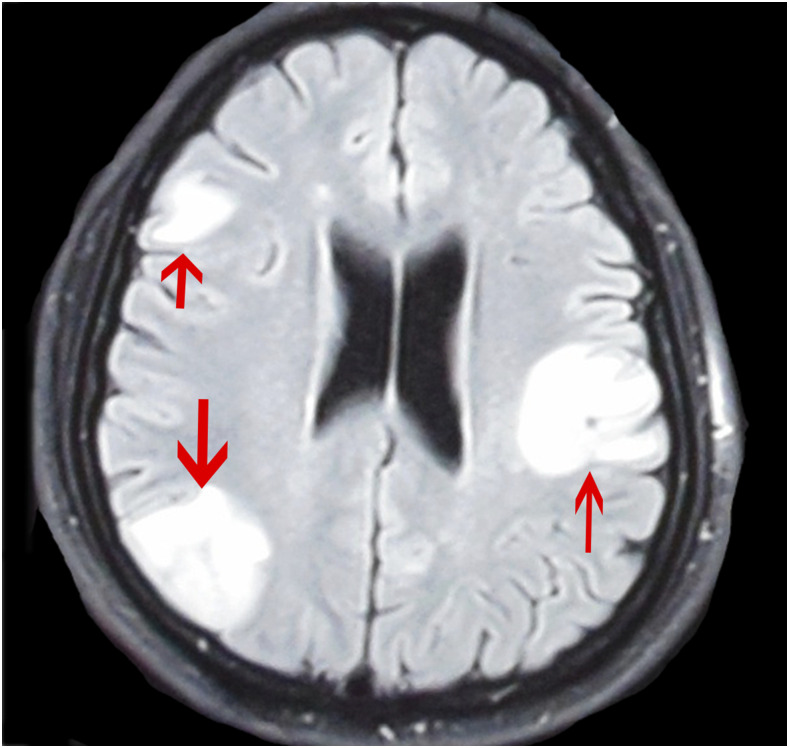
Persistent edema on imaging studies. A 6-month posttreatment axial T2-FLAIR MRI scan from a patient with neurocysticercosis. The scan shows persistent edema in multiple areas, indicated by arrows. This figure appears in color at www.ajtmh.org.

To date, no biomarkers have been identified that can successfully predict which NCC patients with acute seizures will develop epilepsy. However, several imaging findings have shown promise in identifying those at increased risk for treatment-resistant epilepsy, as it occurs in up to 30% of subjects with NCC.^18,21,26,33,51,52^ These imaging findings include 1) an increased number of total, degenerating, and calcified lesions at baseline^18^ (Figure 2), 2) persistent perilesional edema on follow-up imaging studies^53^ (Figure 3), and 3) higher perilesional T2 values (likely indicative of gliosis)^54^ on posttreatment magnetic resonance imaging (MRI) scans. One potential drawback of perilesional edema as a predictive factor for persistent seizures is that this finding could either be caused by vasogenic edema from a seizure or could be due to immune-associated inflammation. One strategy to address this could be to reimage with MRI after a period of at least 2 months following a known seizure to evaluate edema persistence. This would not completely rule out that perilesional edema is due to seizures, however, as the patient could have subclinical ictal activity. A recent study examining calcified NCC lesions with perilesional edema using PET scans found that 12 of 13 new NCC lesions (including both degenerating cysts and areas of perilesional edema, 92.3%) expressed immune-mediated inflammatory proteins, which remained present from 2 to 9 months following treatment.^55^ Future studies using this technology could, therefore, help better understand the pathophysiology of perilesional edema.

### Importance of the immune inflammatory response in epilepsy development.

In both porcine models of NCC and PET studies in humans, lesions of NCC showed evidence of perilesional inflammation.^55,56^ This inflammatory response in NCC appears to play an important role in epileptogenesis, natural involution of lesions, and response to treatment. Inflammation is thought to lead to epileptogenesis either by causing gliosis and/or by causing persistent areas of blood–brain barrier dysfunction.^39^

As evidence to support an association between the inflammatory response and epileptogenesis, subjects with active epilepsy due to NCC, compared with infected subjects without epilepsy, have 1) higher serum levels of blood–brain barrier breakdown molecules (MMP-9) and increase in pro-inflammatory cytokines (tumor necrosis factor [TNF]-α, interferon-γ, and interleukin [IL] 1-β), 2) increased expression of lymphocyte adhesion molecules, and 3) increased likelihood of mutations in the Toll-like receptor 4 that lead to an increased Th1 (pro-inflammatory) response (Figure 1).^19,20,57,58^ Finally, a strong serologic response (≥ 4 bands)^8,59^ to parasite antigen on NCC EITB was associated with both epilepsy development and with a more severe course once epilepsy was established.^18,60^

Given the importance of inflammation in epilepsy due to NCC, several studies have evaluated for a clinical utility of glucocorticoids such as prednisone and dexamethasone to reduce seizures or inhibit epileptogenesis. Steroids have been shown to reduce the incidence of acute seizures in NCC^61,62^ and may be able to impact disease progression.^63^ Steroids have proven beneficial in reducing the frequency of adverse events among subjects with 1) viable intraparenchymal lesions who are treated with antiparasitic medications,^64^ 2) single enhancing or multicystic lesions,^65^ 3) subarachnoid or spinal disease, and 4) those who require ventriculoperitoneal shunt placement.^66^ However, the lengthy treatment courses and high doses of steroids often required to control inflammation associated with NCC can lead to significant side effects, including hyperglycemia, avascular necrosis of joints, and glaucoma.^67^ Newer anti-inflammatory medications such as methotrexate and TNF-α inhibitors likely merit further study to assess their ability to suppress inflammation with potentially fewer side effects than steroids. Although data are limited, one case series included four patients with episodes of perilesional edema and seizures, all of whom reported clinical improvement after treatment with 25 or 50 mg of etanercept weekly for a median of 400.5 days.^68^ Further studies should be carried out with a larger number of patients to confirm these findings.

### The role of calcified lesions and hippocampal sclerosis in epilepsy due to NCC.

Epilepsy rates have been shown to be higher in areas where NCC calcifications (the most frequent finding of NCC^23,69^) are common.^26,70^ Calcium is known to be neurotoxic, and other diseases with neurologic calcifications are also associated with epilepsy. In a select number of patients with cerebral calcifications (with causes including a calcified cavernous hemangioma or idiopathic calcification), the bisphosphonate drug disodium etidronate was found to decrease seizure recurrences, presumably by chelating solid-phase calcium phosphates.^22^ However, this has not yet been studied in calcified NCC lesions.

**Figure 4.**
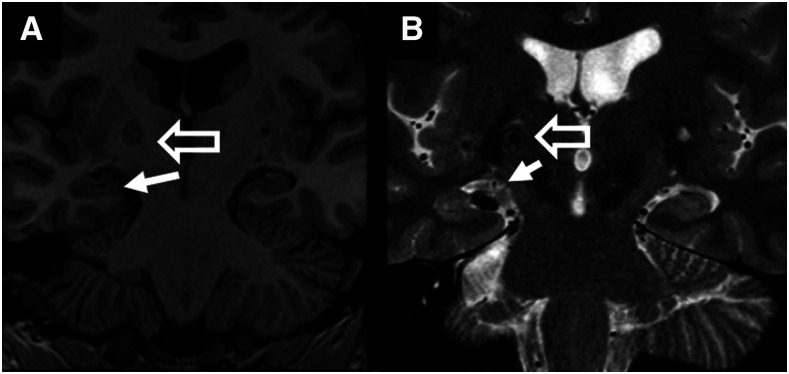
Hippocampal sclerosis. Hippocampal sclerosis (defined as the presence of hippocampal atrophy and a hyperintense signal on long-repetition-time sequences of the hippocampus) is seen on the R-side (solid arrow) of the T1 (**A**) and T2-FLAIR (**B**) coronal MRI scans. An adjacent calcified lesion (hollow arrow) is also shown.

As the neuroanatomic locations of calcified NCC lesions do not always correlate with areas of abnormality on electroencephalogram (EEG), some researchers have suggested that calcifications may be only an incidental finding in epilepsy patients.^23,47,71^ It has recently been suggested that calcifications may be associated with hippocampal sclerosis^71–74^ (HS; a known cause of temporal lobe epilepsy,^75,76^ Figure 4), which could possibly explain this lack of concordance between NCC lesions and EEG abnormalities. Hippocampal sclerosis (HS) is defined as the presence of both hippocampal atrophy and visible sclerosis on MRI.^77,78^ NCC could lead to HS through two possible mechanisms: 1) the NCC lesion could cause chronic interictal or ictal discharges that injure and kindle the hippocampus or 2) NCC-associated inflammation could directly damage the hippocampus.^79^

As evidence for a causative association between calcifications and HS, among patients presenting for an evaluation for epilepsy surgery, patients with HS were less likely to have another precipitating injury of epilepsy identified if they had NCC calcifications (4 of 18 subjects, 22.2% had another injury) compared with those with HS alone (19/36, 52.8%, *P*-value < 0.05).^80^ In addition, in a case–control study, patients with NCC had decreased hippocampal volumes compared with control subjects. On a subgroup analysis (of control subjects, subjects untreated for NCC, and treated NCC subjects), this decrease in hippocampal volumes among NCC patients was found to be primarily due to changes among untreated patients. Mean hippocampal volumes in this study were 3.44 cm^3^ on the right and 3.38 on the left for untreated NCC patients compared with 3.92 cm^3^ and 3.84, respectively, for control subjects (*P* < 0.001).^81^ Furthermore, in a cohort of patients surgically treated for mesial temporal lobe epilepsy, among patients with a single calcified NCC lesion, the lesion matched the side of the HS in 43 of 58 cases (74.1%), significantly more than would be expected because of chance (*P*-value = 0.008).^82^

**Figure 5.**
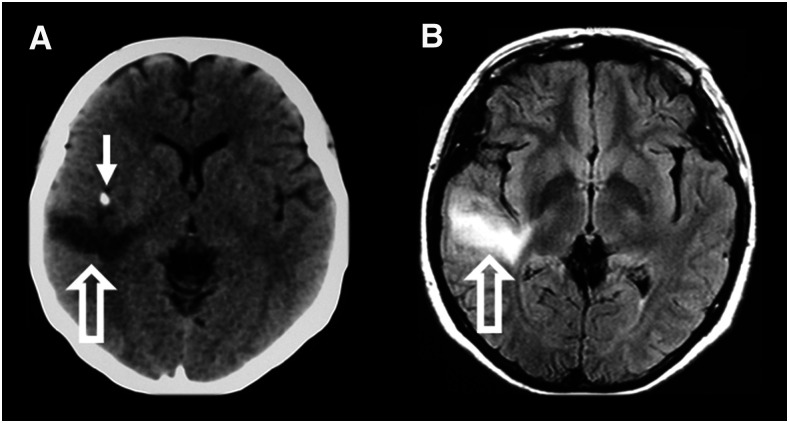
Inflamed calcifications. CT scan (**A)** and concurrent T2-FLAIR MRI scan (**B**) demonstrating axial views of a calcified lesion with perilesional edema seen in the right temporal lobe of both the CT scan and the MRI scan. The calcified lesion is indicated by a solid arrow. Areas of perilesional edema are indicated by hollow arrows.

Recent evidence suggests that calcifications may also lead to epilepsy through an immune inflammatory mechanism. NCC calcifications had long been thought of as immunologically inert, until Nash et al.^83^ demonstrated that calcified lesions of NCC could be associated with perilesional edema (inflamed calcifications, Figure 5). The presence of an inflamed calcification is associated with up to a 12-fold increased likelihood of ongoing seizures despite treatment with anticonvulsants.^18,83–85^ As evidence for an immune etiology of perilesional edema (as opposed to vasogenic edema), pathologic examination of a resected calcified NCC granuloma with recurrent episodes of perilesional edema showed a marked mononuclear infiltrate.^25^ Despite the immunologic causes that appear to underlie inflamed calcifications, the utility of corticosteroids or other immunosuppressive medications in this setting has not been established. Corticosteroid withdrawal has been shown to initiate (or exacerbate) pericalcific edema,^86^ and therefore, currently there is no sufficient evidence that the benefits of steroids outweigh the risks.

Because of the focal nature of NCC lesions, a promising option for a subset of patients with medically refractory epilepsy and NCC has been surgical resection. Although a minority of NCC patients would likely qualify for such treatment, patients may benefit from surgery if they have 1) intractable epilepsy with an NCC lesion within the boundaries of the epileptogenic zone or 2) developed HS in conjunction with NCC. In two small reviews,^87,88^ 16 subjects who had calcified NCC lesions plus either evidence that a specific calcified lesion was the seizure-provoking focus (based on seizure semiology and/or EEG findings) or association of the calcification with unilateral HS were treated surgically. Those without evidence of HS underwent resection of the causative lesion, whereas those with HS were treated with resection of both the calcified lesion and the anterior temporal lobe (ATL). Following surgery, 93.8% (15/16) of subjects experienced complete resolution of their seizures. However, resection of solely the NCC lesion or the ATL alone was not found to be successful in subjects with both HS and a causative lesion due to NCC (4/5 subjects, 80%, continued to have seizures). Although surgical resection has been promising in this small group of patients, which surgical technique is best and what subset of patients are most likely to benefit from such an intervention are not yet known.

## CONCLUSION

Recent studies have identified two primary processes, inflammation and calcification (in association with HS or not), which appear to be important for epilepsy development in patients with NCC. Factors associated with these processes (such as quantitative edema, an increased number of calcifications, and the presence of calcified lesions with perilesional edema) may be able to identify those at increased risk for severe seizure disorders early in their disease course. Both of these processes could potentially be treated with currently available medications that could be repurposed for use in NCC (either steroid-sparing anti-inflammatory medications such as methotrexate or etanercept or bisphosphonates). Studies of these treatments in NCC patients are needed as a large portion of those with NCC and epilepsy continue to seize despite treatment.
